# Knowledge, attitudes and demographic effect on menopausal experiences among Indian rural women

**DOI:** 10.6026/973206300200175

**Published:** 2024-02-29

**Authors:** N Sivasubramanian, Chaudhari Pinalben Madhabhai, Amita Shilpa Gottlieb, B Mahalakshmi, Payal Vaghela, Sandeep Garg

**Affiliations:** 1 Department of Psychiatric Nursing, Nootan College of Nursing, Sankalchand Patel University, Visnagar, Gujarat - 384315, India; 2 Department of Pediatric Nursing, Nootan College of Nursing, Sankalchand Patel University, Visnagar, Gujarat - 384315, India; 3 Department of obstetric and gynaecological Nursing, Graphic Era College of Nursing, Graphic Era Deemed to be University, Dehradun, Uttarakhand -248002, India; 4 Nootan College of Nursing, Sankalchand Patel University, Visnagar, Gujarat - 384315, India; 5 Department of Community health Nursing, College of Nursing, S.G.R.R University, Dehradun, Uttarkhand - 248001, India; 6 Mewar Bsc Nursing College, Udaipur, Rajasthan - 313001, India

**Keywords:** Menopause, rural women, menopausal experiences, knowledge

## Abstract

The menopausal experiences of women in selected rural areas of Visnagar, addressing a research gap within cultural and socio-economic landscapes is of interest. The global aging trend was reflected in the 26% representation of women aged 50 and over in the study. Employing a cross-sectional design, 200 menopausal women were sampled through stratified random sampling, emphasizing demographic diversity. Results from a structured Knowledge Questionnaire and a Likert Scale for Attitude revealed that women generally possessed commendable knowledge (mean score 10.94) and positive attitudes (mean score 28.66) toward menopause. Significant associations between age, education, occupation, and income with knowledge and attitudes were uncovered through chi-square tests. This study underscored the need for tailored interventions in rural settings, considering the influence of cultural, regional, and demographic factors on menopausal experiences. The findings contributed to bridging the research gap and emphasized the importance of individualized approaches for promoting the well-being of menopausal women in rural communities.

## Background:

The global population of postmenopausal women is growing. In 2021, women aged 50 and over accounted for 26% of all women and girls globally [1]. Globally, a woman aged 60 years in 2019 could expect to live on average another 21 years [2]. Menopause, a natural biological milestone, symbolizes a pivotal phase in a woman's life, typically manifesting in her late 40s or early 50s. This transformative journey marks the cessation of menstrual cycles, accompanied by a diverse array of physical and emotional changes [3]. The universality of menopause is underscored by the fact that it is an experience shared by women worldwide. However, the nuanced nature of menopause experiences is deeply rooted in the individual's cultural background, level of education, and socio-economic status [4]. This complex transition encompasses a spectrum of physical and emotional changes that can impact a woman's overall well-being. The physical manifestations range from hormonal fluctuations to alterations in bone density and cardiovascular health [5]. Concurrently, the emotional dimensions may involve mood swings, sleep disturbances, and changes in libido. Acknowledging the intricate interplay of these factors is crucial for addressing the holistic health needs of women undergoing menopause [6]. Current data underscores the imperative of understanding menopause due to its profound implications for women's health and well-being. Various studies highlight discernible gaps in knowledge and attitudes toward menopause, emphasizing the necessity for investigations that consider the specific contexts in which women experience this life stage [7]. The recognition of these gaps serves as the impetus for our study, which focuses on menopausal women residing in selected rural areas of Visnagar. The rationale for this research lies in the unique challenges faced by women in rural settings, where access to information and healthcare resources is often constrained [8]. Cultural norms and limited educational opportunities further contribute to shaping perceptions and practices related to menopause. By delving into this specific context, we aim to unravel the intricacies of menopause experiences in rural Visnagar, providing insights crucial for tailored healthcare interventions. This study holds significance in addressing the dearth of comprehensive research in understanding menopause within the cultural and socio-economic landscape of rural areas. By examining the interplay between knowledge, attitude, and demographic variables, we seek to identify patterns that can inform targeted interventions, ultimately promoting the holistic well-being of menopausal women in these specific rural communities.

## Methodology: 

## Research design:

A cross-sectional research design is employed to capture a snapshot of menopausal women's knowledge and attitudes in selected rural areas of Visnagar.

## Research setting:

The study is conducted in specific rural areas of Visnagar, chosen for their representativeness and demographic diversity.

## Sample size and sampling technique:

The sample size comprises 200 menopausal women, selected through stratified random sampling to ensure diversity in age, education, occupation, and socioeconomic status [9].

## Data collection tool:

Data is collected using a structured Knowledge Questionnaire and a Likert Scale for Attitude, aiming to comprehensively assess knowledge and attitudes related to menopause.

## Data collection procedure:

Face-to-face interviews conducted by trained research assistants in private settings ensure reliable responses while maintaining participant confidentiality.

## Ethical considerations:

Ethical approval is obtained, and informed consent is secured from participants, emphasizing voluntary participation, confidentiality, and the right to withdraw.

## Statistical analysis plan:

Descriptive statistics include mean, standard deviation, frequencies, and percentages. Inferential statistics, such as chi-square tests, explore associations, with a significance level set at p < 0.05.

## Results:

**Figure 1:**
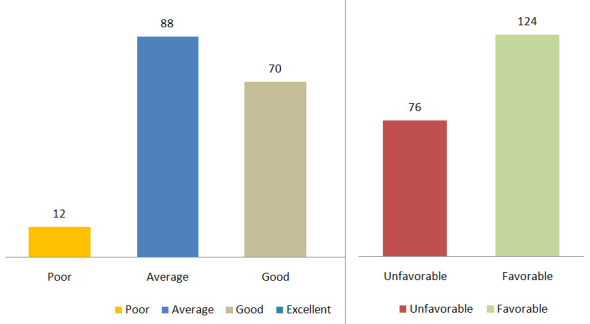
Bar graph showing distribution of mother as per their knowledge and attitude score

Table 1 summarizes key demographic characteristics of the 200 menopausal women (Figure 1). Predominantly aged 45-50, the sample is largely Hindu homemakers with secondary education. Most belong to nuclear families, have two children, and follow a vegetarian diet. Marital status is predominantly married, and the majority have prior knowledge of menopause from sources like mass media and health personnel.Regarding knowledge score, mean and standard deviation are (10.94±4.09), and the attitude score mean and standard deviation are (28.66±6.27) for the sample regarding menopause. The results indicated that 6% had poor knowledge, 44% had average knowledge, 35% had good knowledge, and 15% had excellent knowledge. Subsequently, an analysis of attitudes toward menopause revealed that 38% held an unfavorable attitude, while 62% had a favorable perspective, as depicted. Table 2 unveils specific associations between demographic variables and both knowledge and attitude scores among menopausal women in selected rural areas of Visnagar. In the realm of knowledge, significant associations are observed with age and educational status. Younger age groups and higher educational levels correlate positively with enhanced knowledge. Occupation plays a distinctive role, delineating knowledge disparities among different occupational groups. Religion, marital status, number of children, types of food, and previous knowledge, however, do not exhibit significant associations with knowledge scores. Turning to attitudes, age and educational status again surface as influential factors. Younger age groups and higher educational levels are associated with more positive attitudes toward menopause. Occupation significantly influences attitudes, showcasing variations across occupational groups. Religion, marital status, number of children, types of food, and previous knowledge do not exhibit significant associations with attitude scores. This detailed breakdown illuminates the intricate relationships between demographic variables and both knowledge and attitude scores among menopausal women in rural Visnagar.

## Discussion:

Data shows that the mean knowledge score of 10.94 signifies a commendable level of menopausal understanding among women in rural Visnagar. A similarly positive trend in knowledge was observed among women in a distinct cultural context [10]. In contrast, a study conducted by Monika *et al.* (2021) reported lower knowledge levels, emphasizing the variability in menopausal awareness across diverse populations [11]. Regarding attitudes, the mean attitude score of 28.66 in our study suggests generally positive sentiments toward menopause. This resonates with the findings of Diana *et al.* [2008), which reported a prevalent positive attitude among menopausal women in an urban setting [12]. However, the current study's variability in individual attitudes, indicated by a standard deviation of 6.27, contrasts with the more uniform attitudes reported by Ayer *et al.* (2010), underlining the need for tailored interventions based on individual differences [13]. Demographic associations such as age and educational status emerged as influential factors as shown elsewhere [14,15]. However, our emphasis on the impact of occupation and income provides additional context specific to the rural setting in Visnagar. This contrasts Helen Gebretatyos *et al.* (2020), where occupation showed limited influence, emphasizing the importance of considering regional variations in demographic associations [16,17]. These comparisons underscore the dynamic nature of menopausal experiences, influenced by cultural, regional, and demographic factors. While certain patterns are similar with previous studies, unique findings highlight the importance of tailoring interventions to the specific needs of women in rural Visnagar.

## Conclusion:

Data shows that the menopausal experiences of women in rural Visnagar, emphasizing demographic influences on knowledge and attitudes. The global trend of an aging population is mirrored in the study's 26% representation of women aged 50 and over. The methodology captures a snapshot of 200 women, revealing significant associations between age, education, occupation, and income with knowledge and attitudes. Findings contribute to addressing the research gap in understanding menopause within rural contexts, providing insights for tailored interventions. The study underscores the dynamic nature of menopausal experiences influenced by cultural, regional, and demographic factors. Ultimately, it emphasizes the importance of individualized approaches for promoting the well-being of menopausal women in rural communities.

## Figures and Tables

**Table 1:** Description of mother as per demographic characteristics

**Characteristics**	**Frequency**	**Percentage**
Age		
45-50	110	55%
51-55	40	20%
56-60	29	14.50%
61-65	21	10.50%
Educational qualification		
Illiterate	30	15%
Primary	22	11%
Secondary	68	34%
High secondary	33	16.50%
Graduate	27	13.50%
Post-graduate and above	20	10%
Religion		
Hindu	196	98%
Muslim	4	2%
Christian	0	0%
Others	0	0%
Occupation		
Private	18	9%
Government	22	11%
Homemakers	162	81%
Others	8	4%
Types of family		
Joint	40	20%
Nuclear	152	76%
Extended	8	4%
Number of children		
Nil	0	0%
One	42	21%
Two	112	56%
>Two	46	23%
Types of food		
Vegetarian	190	95%
Non-vegetarian	10	5%
Total monthly income		
5,000	0	0%
5,000-10,000	55	27.50%
10,001-25,001	70	35%
>15,000	75	37.50%
Marital status		
Unmarried	0	0%
Married	195	97.50%
Widow	2	1%
Divorced	3	1.50%
Previous knowledge regarding menopause		
Yes	192	96%
No	8	4%
Source of information		
Mass-media	76	38%
Health-person	72	36%
Relatives	44	22%
Others	0	0%

**Table 2:** Association of knowledge score and attitude score with selected demographic variables

**Sr. No.**	**Demographic Variable**	**attitude**	**Knowledge**
		**Chi Square Result**	**Chi Square Result**
1)	Age of Mother (in years)	27.15 Significant (S)	25.50 Significant (S)
2)	Educational Status	19.04 Significant (S)	23.12 Significant (S)
3)	Religion	0.24 Non-Significant (NS)	3.17 Non-Significant (NS)
4)	Occupation	11.12 Significant (S)	37.02 Significant (S)
5)	Types of Family	3.86 Non-Significant (NS)	93.73 Significant (S)
6)	Number of Children	3.48 Non-Significant (NS)	76.51 Significant (S)
7)	Types of Food	0.16 Non-Significant (NS)	4.37 Non-Significant (NS)
8)	Total Monthly Income	5.18 Non-Significant (NS)	74.73 Significant (S)
9)	Marital Status	0.329 Non-Significant (NS)	1.05 Non-Significant (NS)
10)	Previous Knowledge Regarding Menopause?	2.32 Non-Significant NS)	47.47 Non-Significant (NS)
11)	First Source of Information	5.41 Significant (S)	2.12 Non-Significant (NS)
